# Integrating Structural, Biochemical, and Cellular Perspectives on the TFIIH Helicases XPB and XPD

**DOI:** 10.3390/biom16030435

**Published:** 2026-03-13

**Authors:** Marco Bravo, Li Fan

**Affiliations:** Department of Biochemistry, University of California, 900 University Ave, Riverside, CA 92521, USA; marco.bravo@ucr.edu

**Keywords:** DNA repair, nucleotide excision repair, transcription, genome maintenance, triptolide, spironolactone, cancer

## Abstract

Xeroderma pigmentosum group B (XPB/ERCC3) and group D (XPD/ERCC2) helicases are integral components of the transcription factor IIH (TFIIH) complex, coordinating DNA unwinding during transcription initiation and nucleotide excision repair (NER). XPB functions as an ATP-driven translocase that generates torsional strain to promote promoter melting and DNA opening at lesion sites, whereas XPD acts as a 5′ to 3′ helicase responsible for lesion verification and extension of the repair bubble. Structural and biochemical studies have clarified how TFIIH subunits regulate these helicases—p52 and p8 modulate XPB’s translocation activity, while p44, p62, and MAT1 control XPD’s helicase function through conformational and compositional transitions within the complex. Beyond their canonical roles, XPB and XPD participate in diverse cellular pathways, including cell-cycle regulation and oxidative stress response, highlighting their involvement in maintaining genome integrity beyond repair and transcription. Mutations in either helicase lead to xeroderma pigmentosum (XP), trichothiodystrophy (TTD), or combined XP/Cockayne syndrome (XP/CS) phenotypes, emphasizing the essential role of TFIIH integrity for human health. Recent biochemical and pharmacological advances have further revealed the therapeutic relevance of these helicases—XPB as a target of small-molecule inhibitors such as triptolide, Minnelide, and spironolactone, and XPD as a potential modulator of cancer sensitivity to DNA-damaging treatments. Collectively, XPB and XPD exemplify the structural and functional versatility of TFIIH helicases across repair, transcription, and genome maintenance.

## 1. Introduction

Living cells continuously face DNA damage from ultraviolet radiation, reactive oxygen species, and chemical mutagens that threaten genomic integrity. To counteract such lesions, eukaryotic cells rely on multiple repair pathways, among which NER uniquely removes bulky, helix-distorting adducts that impede transcription and replication [1,2]. NER proceeds through sequential stages of lesion recognition, local DNA unwinding, dual incision, and resynthesis of the excised fragment, ensuring accurate restoration of the DNA duplex [2]. Defects in this process lead to a spectrum of human disorders, including XP, CS, and TTD, each illustrating the importance of NER in maintaining genome stability and transcriptional fidelity [3,4,5,6].

**Figure 1:**
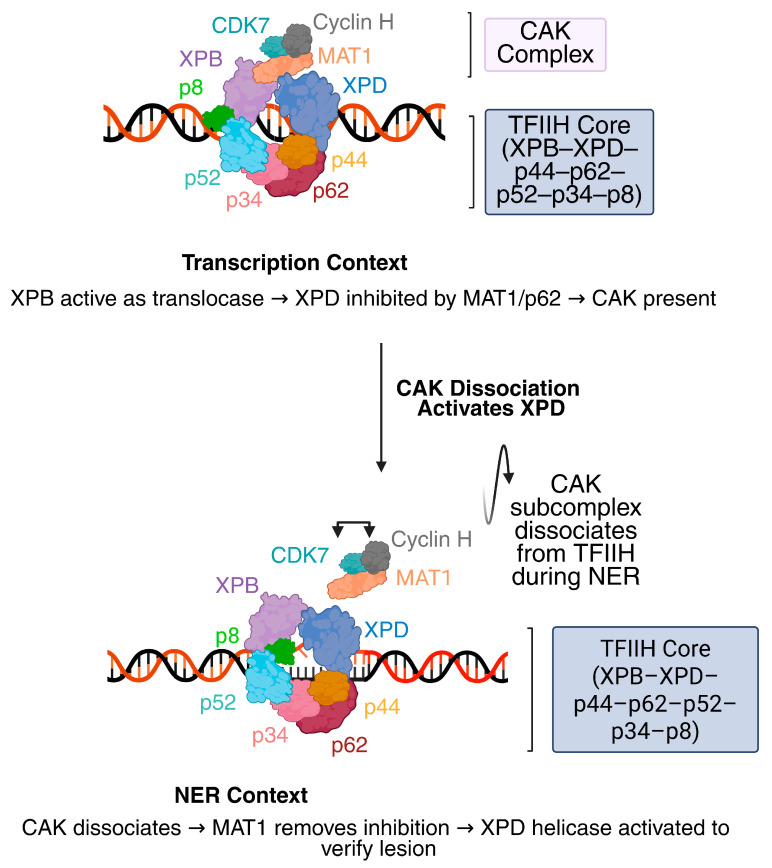
Different TFIIH conformations in transcription versus NER. During transcription initiation (upper panel), TFIIH adopts a transcription-compatible conformation in which the CAK subcomplex (CDK7–Cyclin H–MAT1) remains associated with the TFIIH core (XPB, XPD, p44, p62, p52, p34, p8/TTDA). In this state, XPB functions as the active ATP-dependent dsDNA translocase within TFIIH, whereas XPD helicase activity is restrained. MAT1 binds the XPD ARCH domain, maintaining XPD in a helicase-inactive configuration. The core subunits stabilize this arrangement: p52 interacts with the XPB N-terminal region; p34 contributes to structural integrity through interactions within the XPB–p52 interface; and p62 associates with XPD within the core complex. Together, these interactions maintain a transcription-compatible organization in which XPB is active and XPD is inactive. Upon NER activation (lower panel), the CAK subcomplex dissociates from TFIIH, relieving MAT1-mediated inhibition of XPD. This permits conformational rearrangement of the TFIIH core and repositioning of XPD into a repair-competent state capable of engaging DNA. In this NER-specific configuration, productive interaction between XPD and p44–p62 supports helicase activity and XPD translocation during lesion verification. XPB remains associated with the downstream duplex and contributes to DNA opening during repair-bubble formation. The figure was adapted from [10]. Models were created in BioRender. Bravo, M. (2026) https://BioRender.com/6rdgf03.

At the center of both NER repair and transcription initiation is the TFIIH complex, a ten-subunit assembly required for DNA opening in both processes. Within this complex, XPB and XPD provide the enzymatic activities that mediate DNA unwinding [7]. XPB functions as an ATP-dependent double-stranded DNA translocase that contributes to promoter opening during transcription initiation and to DNA bubble formation during NER. XPD acts as a 5′ → 3′ helicase that binds single-stranded DNA within the repair bubble and participates in lesion verification [7]. Together XPB and XPD create a repair bubble of approximately 23–25 nt in size prior to incision [7]. Their enzymatic activities are modulated by auxiliary TFIIH subunits, with p52 and p8 regulating XPB, and p44, p62, and MAT1 regulating XPD during transcription and repair (Figure 1) [8,9].

The organization of TFIIH reveals a coordinated mechanism in which XPB and XPD function as complementary motor proteins during DNA repair [11]. Structural and biochemical studies describe XPB-driven translocation as the initiating step of local DNA opening, followed by XPD-mediated scanning and verification of damaged sites [12]. Cryo-electron microscopy has captured TFIIH conformational transitions that support this sequential activation model and highlight how coordinated domain movements within the complex facilitate DNA engagement and lesion recognition [12,13].

This review synthesizes structural and biochemical evidence defining the roles of XPB and XPD helicases within TFIIH, emphasizing how their coordinated actions promote DNA opening during NER and transcription. By integrating insights from eukaryotic and archaeal systems, it examines shared mechanisms, divergent functional adaptations, and aspects regarding TFIIH dynamics and disease-associated dysfunction.

## 2. XPB

The classification of XP cells into complementation groups XP-A through XP-G was originally established through cell fusion assays that identified distinct genetic defects associated with UV sensitivity in fibroblasts, among which XPB represents the helicase-deficient XP-B group [14]. XPB, also known as ERCC3, is a conserved superfamily 2 (SF2) DNA helicase that functions as one of the ten subunits of TFIIH, which is essential for transcription and NER (Figure 1) [1]. XPB functions as an ATP-dependent DNA translocase within the TFIIH complex, where it unwinds DNA around the lesion’s 5′ side during NER and promotes promoter opening during RNA polymerase II transcription initiation by generating the transcription bubble required for start-site melting [2].

**Figure 2:**
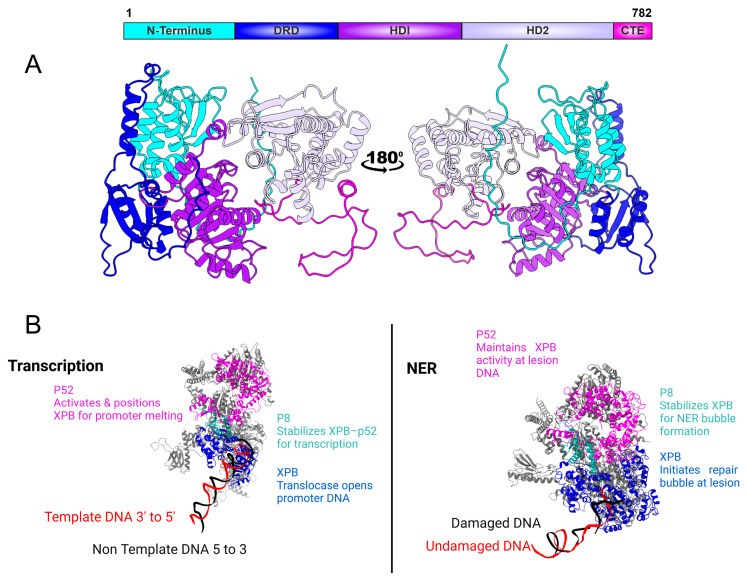
Structure of human XPB and its different positionings during transcription and NER. (**A**). Domain organization of human XPB and its 3D structure predicted by AlphaFold3. (**B**) Differential positioning of XPB (blue), p52 (purple), and p8 (cyan) within TFIIH during transcription and NER. Structural comparison of TFIIH highlights distinct conformational arrangements of XPB and its regulatory subunits p52 and p8 in transcription (PDB ID: 5OQJ) (**left**) versus NER (PDB ID: 7K04) (**right**). During transcription initiation, p52 activates and positions XPB to function as a DNA translocase that drives promoter melting, while p8 stabilizes the XPB–p52 interaction to support open complex formation. XPB engages promoter DNA, promoting strand opening in a defined 3′–5′ direction relative to the template and non-template strands. In contrast, during NER, TFIIH undergoes repositioning such that p52 and p8 maintain and stabilize XPB activity at sites of DNA damage. XPB initiates repair bubble formation at the lesion, enabling subsequent damage verification and processing. DNA strands and key subunits are color-coded while the remaining components are shown in gray for clarity; only XPB, XPD, p44, p62, p52, p8, and DNA are retained (with MAT1 additionally retained in 5OQJ).

XPB exists within all three domains of life and shares a similar overall fold and architecture, although the function of XPB in the archaea and bacteria is more elusive [15,16,17]. Initial structural studies on archaeal XPB revealed, for the first time, that the protein contains two RecA-like helicase domains (HD1 and HD2) and an N-terminal damage recognition domain (DRD) [18]. Cryo-EM analysis of human TFIIH demonstrated that XPB retains the conserved RecA-like helicase architecture observed in archaeal homologs and includes an additional N-terminal extension that interacts with p52 within the TFIIH core (Figure 2) [11]. Eukaryotic XPB also contains a C-terminal extension that includes Ser751, whose phosphorylation negatively regulates lesion incision by the NER nuclease ERCC1–XPF [11,19,20,21].

In plants, XPB function exhibits lineage-specific diversification. *Arabidopsis thaliana* contains two tandemly duplicated XPB genes, AtXPB1 and AtXPB2, a duplication event restricted to the Camelineae lineage [22]. Expression profiling revealed that AtXPB2 clusters with other TFIIH subunits in proliferating tissues, while AtXPB1 is selectively induced by UV-B exposure in leaf trichomes. These non-overlapping patterns support subfunctionalization, with AtXPB2 contributing broadly to transcription-coupled processes and AtXPB1 acting in specialized DNA repair contexts [22].

### 2.1. XPB’s Role in Transcription

Biochemical reconstitution of the RNA polymerase II preinitiation complex showed that XPB functions as a double-stranded DNA translocase rather than a conventional helicase during transcription initiation [23]. XPB tracks along the non-template strand in the 5′ to 3′ direction, reeling downstream promoter DNA into the RNA polymerase II cleft and generating torsional strain that drives promoter unwinding [23]. Blocking XPB translocation on the non-template strand inhibited transcription initiation, confirming that its ATP-dependent translocation is essential for formation of the open complex, defined as the locally unwound promoter state that creates a transcription bubble permitting template strand access, and for promoter escape, the transition in which RNA polymerase II clears promoter contacts and proceeds into productive elongation [23]. The processivity of XPB was limited to roughly one turn of DNA, consistent with the transient nature of this open promoter state and the requirement for continuous ATP hydrolysis to maintain promoter opening [23].

Mutational analysis showed that conserved regions of TFIIH, including Ssl2 (XPB in yeast), influence transcription start site distributions by determining how far promoter DNA can be scanned before initiation [24]. Distinct Ssl2 allele classes produced genome-wide changes in initiation patterns consistent with either shortened or extended scanning ranges [24]. Genetic interactions with other initiation factor mutants further differentiated these allele classes and supported their distinct functional behaviors [24]. Cryo-EM structures of the yeast preinitiation complex revealed that Ssl2 is positioned about 25–30 base pairs downstream of the transcription start site, bound to downstream duplex DNA [25]. XPB adopts a bilobal ATPase configuration typical of superfamily 2 helicases, with both lobes contacting the DNA backbone [25]. A C-terminal extension from lobe 2 interacts with Tfb5, the yeast homolog of human TTDA (GTF2H5 or p8), a small structural subunit that stabilizes the TFIIH core and helps maintain Ssl2’s orientation during DNA engagement [25]. Additional interactions with Tfb2, the homolog of human p52 (GTF2H4), further secure Ssl2 within the complex [25]. The “E-bridge” helix of TFIIE contacts lobe 2 of XPB, consistent with TFIIE-stimulated ATPase activity [25]. In the +1 nucleosome–bound PIC (preinitiation complex)–Mediator structure, XPB was observed to contact promoter DNA at +11/+21 and to engage nucleosomal DNA through a positively charged patch, forming part of an interaction network that may stabilize PIC–Mediator on chromatin [26].

High-resolution mammalian PIC structures provided direct visualization of XPB during the early steps of promoter opening [27]. XPB was captured in both pre- and post-translocation ATPase states, revealing how ATP binding closes the two RecA-like lobes to advance the non-template strand by one base pair, while ATP hydrolysis reopens the lobes for the next step [27]. This cycle induces progressive twisting of downstream promoter DNA, supplying the mechanical torque that initiates bubble formation and drives the transition from the closed complex (CC) to the open complex (OC) [27]. The structures also showed that XPB undergoes a pronounced positional shift upon PIC assembly: contacts with MAT1 are released, its interface with p52 is rearranged, and the motor is repositioned so its translocase activity is aligned with the downstream duplex [27]. These rearrangements distinguish the transcriptional form of TFIIH from its repair form and structurally explain how XPB becomes an active dsDNA translocase within the PIC capable of driving the CC → OC transition [27].

In the fully assembled holo preinitiation complex, TFIIH is positioned with XPB grasping the downstream promoter DNA for promoter opening [28]. The horseshoe-shaped TFIIH core has XPB and XPD at opposite ends bridged by MAT1’s N-terminus [28]. These structures identify XPB as the central translocase driving promoter opening within the TFIIH core. High-resolution cryo-EM analysis of the human TFIIH core complex revealed how XPB is anchored to the p52 and p8 subunits through a pseudo-symmetric interface between the XPB NTD and the p52 “clutch” domain [29]. This architecture explains how p52 and p8 contribute to XPB recruitment and stimulation of its ATPase activity within TFIIH [29].

Structural comparisons suggest that XPB can undergo conformational changes upon incorporation into the RNA polymerase II preinitiation complex, positioning it for DNA translocation during promoter opening [29]. Mapping of disease-associated mutations such as F99S and T119P indicates that they destabilize XPB folding and its interaction with p52, potentially affecting TFIIH assembly and transcriptional activity [29]. Structural and mutational analyses have further defined the interaction between XPB and its regulatory partner p52 [30]. Alanine-scanning mutagenesis of the conserved HubA domain of yeast Tfb2, the p52 homolog, revealed that hydrophobic and electrostatic contacts within this region are essential for anchoring XPB/Ssl2 to TFIIH [30]. Disruption of this interface destabilized the complex and reduced transcriptional activity, indicating that p52 not only recruits XPB but also stabilizes it in a conformation required for DNA translocation during promoter opening [30].

XPB levels are tightly regulated in vivo, with each cell type maintaining a characteristic and highly uniform XPB concentration [31]. This strict control likely reflects XPB’s rate-limiting role within TFIIH during transcription initiation [31]. Using an Xpby/y mouse model, a study showed that steady-state XPB abundance closely correlates with basal transcriptional activity and proliferative status, establishing the model as a functional biomarker system for assessing transcriptional output and for screening or toxicological evaluation of small molecules [31].

Interestingly RNA polymerase II initiation for some genes can occur without XPB [32]. Acute XPB degradation by spironolactone, discussed in detail in Section 2.4, left global mRNA synthesis largely intact, whereas inhibition of its ATPase activity by triptolide, as discussed in Section 2.4, completely blocked transcription [32]. Using 5-ethynyl-uridine labeling, chromatin immunoprecipitation, and in vitro run-off transcription assays, XPB-depleted TFIIH complexes containing XPD, p62, p52, p44, p34, and CDK7 were shown to still assemble at promoters and support initiation, while NER was abolished in the absence of XPB [32]. Mutations within XPB’s RecA-like helicase domains (T469A, Q638A) rendered transcription resistant to triptolide indicating that inhibition of XPB’s ATPase activity prevents transcription only when XPB protein is present [32]. These results support the “built-in block” model proposed in the study, in which XPB intrinsically imposes a block to promoter melting within the pre-initiation complex. Under normal conditions, ATP hydrolysis by XPB relieves this inhibitory constraint, allowing promoter opening to proceed. When XPB is depleted, the block is absent and transcription can occur without XPB ATPase activity. In contrast, when XPB is present but its ATPase activity is inhibited, the block remains in place and transcription is impaired [32]. In this framework, XPB’s ATPase activity functions to release an initiation block imposed by XPB itself rather than to directly unwind DNA as a conventional helicase [32,33].

### 2.2. XPB’s Role in NER

In NER, TFIIH is recruited to the lesion site where XPB provides the ATP-driven activity required to locally open damaged DNA. Acting as a 5′ → 3′ translocase rather than a canonical helicase, it generates torsional strain that promotes DNA unwinding around the lesion [19]. Its activity is finely tuned by the p52 and p8 subunits, which modulate ATPase efficiency and stabilize XPB within the TFIIH core [19]. Through this ATP-dependent motion, XPB anchors TFIIH at the damage site and primes the DNA for XPD-mediated verification and subsequent dual incision [34]. Coin et al. showed that XPB’s ATPase activity, not its helicase function, is required for DNA opening at sites of damage [35]. They identified that the p52 subunit directly interacts with XPB to stimulate this ATPase activity, and disruption of this interaction by the F99S mutation blocked DNA unwinding despite normal TFIIH recruitment. Mechanistically, the study revealed that XPB acts more like a molecular wedge, using ATP-driven conformational changes to keep DNA strands apart and facilitate subsequent unwinding by XPD. In contrast, mutations in helicase motifs III and VI abolished XPB’s helicase activity without impairing repair efficiency, further supporting this novel model of DNA damage repair [35].

Loss of the p8/TTD-A subunit results in decreased XPB stability and partial disassembly of the TFIIH complex, demonstrating that XPB’s maintenance depends on p8 for proper structural integrity [36]. XPB functions as a critical ATPase in NER, using its energy to open DNA around damage sites and remodel the repair complex. Its ATPase activity, stimulated by p8’s first ten N-terminal residues, is essential for repositioning repair factors and unwinding DNA. This dependency is specific to DNA repair, as transcriptional activity remains largely unaffected in p8-deficient cells, highlighting a repair-selective role for p8 in preserving XPB function during NER [36]. XPB’s ATPase activity can be activated by either DNA or the p52/p8 subunits, but p52/p8 impose a dominant limit on XPB even when DNA is present, defining them as regulators that both enable and restrict XPB’s catalytic output [37]. XPB-driven translocase activity occurs only within fully assembled core TFIIH, where XPB promotes DNA opening through ATP-dependent dsDNA translocation rather than conventional helicase-mediated strand separation. XPA enhances the processivity of this complex without altering XPB’s ATPase rate. Together, these findings clarify that p52, p8, and XPA regulate and coordinate XPB’s translocase-driven DNA opening activity during NER [37].

Advances in computational power have enabled molecular dynamics simulations to probe the dynamic behavior of NER complexes, overcoming limitations imposed by static structural models. Through molecular dynamics it was proposed that XPB acts first to unwind DNA downstream of the lesion site, which is recognized by XPC/HR23B/CETN2 and held at the 3′ edge of the expanding DNA bubble [38]. Recruitment of XPA stimulates XPB unwinding by clamping dsDNA and preventing its dissociation from XPB [38]. XPB functions as a translocase, which simultaneously rotates the downstream duplex and pushes it toward XPA [38].

Recent cryo-EM analyses have further clarified XPB’s function in NER. In 2021, van Eeuwen and colleagues reported a cryo-EM reconstruction of the yeast NER initiation complex containing Rad4–Rad23–Rad33 and TFIIH [39]. The structure, which visualized DNA containing a carcinogen-induced lesion, revealed that a ~30-bp duplex extends between Rad4 and the Ssl2 subunit of TFIIH. Simultaneous binding of Rad4 (the yeast homolog of human XPC) and TFIIH was possible because the DNA is locally unwound at the lesion [39]. Cooperative torque generated by Ssl2 and Rad4 binding promotes further DNA opening, exposing the damaged strand for Rad3 (the yeast homolog of human XPD) mediated scanning [39]. These findings emphasize XPB’s central role in lesion-induced DNA unwinding during NER [39].

High-resolution cryo-EM reconstruction of a fully reconstituted human global genome repair assembly capturing all components required for dual incision resolved XPB within the pre-incision complex and delineated its ATPase-coupled structural states [40]. The helicase domains HD1 and HD2 were observed in distinct open and closed conformations, with ATP binding associated with domain closure and release of ADP and phosphate associated with reopening. Structural comparison of these states was correlated with duplex DNA translocation in a two-step cycle in which ATP binding advances one DNA strand by 1 nucleotide and release of ADP and phosphate advances the opposite strand by 1 nucleotide, producing a net movement of 1 base pair per ATP hydrolyzed while base pairing remains intact. The authors refer to this alternating strand advancement during successive phases of the ATPase cycle as a “waddling mechanism,” reflecting sequential duplex progression rather than strand separation [40].

Different XPB variants exhibit distinct structural dynamics, with some helicases like *Archaeoglobus fulgidus* XPB (AfXPB) maintaining a stable open conformation that transforms upon ATP binding [41]. This transformation enables a “molecular wrench” mode of DNA unwinding, which appears to be the primary mechanism for initiating DNA duplex opening at damage sites [41]. The research suggests XPB operates through two complementary modes of action: a rapid molecular wrench mechanism that initially breaks DNA strand base pairing, and a slower conventional unzipping process. These modes are critical for NER, with XPB initiating DNA duplex opening through an unconventional molecular wrench mechanism followed by a slower unzipping process, working together with XPD to extend the DNA bubble for damage incision [41]. A follow up study using similar electrochemical analysis of AfXPB and *Sulfolobus tokodaii* XPB2 (StXPB2) revealed two kinetic modes of helicase activity [42]. At low concentrations, both enzymes showed slow unwinding, while AfXPB at higher concentrations displayed a rapid “molecular wrench” mode nearly three orders of magnitude faster [42]. The endonuclease Bax1, a known XPB-interacting partner, modulated this behavior in opposite ways: AfXPB–Bax1 retained only the slow conventional kinetics, while StXPB2–Bax1 displayed enhanced ATP-stimulated activity [42]. These results demonstrate that Bax1 differentially influences XPB conformational states, stabilizing AfXPB in an inactive open form and promoting StXPB2 closure consistent with catalytic activation [42].

In archaea, the lack of a TFIIH-like assembly leads XPB to associate with Bax1—an XPG-related nuclease—forming a helicase–nuclease complex that coordinates DNA duplex unwinding with strand incision [43,44,45,46]. Structural analysis of archaeal XPB–Bax1 complexes revealed that Bax1 interacts primarily with XPB’s thumb-like (ThM) and HD2 regions through its N-terminal domain. Bax1 modulated XPB’s ATPase activity in a species-dependent manner, enhancing it about fivefold in *Sulfolobus tokodaii* but slightly reducing it in *Archaeoglobus fulgidus* [47]. The complex was further stimulated by Y-shaped and bubble DNA substrates, indicating activation by DNA structures resembling NER DNA intermediates [47].

Structural analysis of the *Sulfolobus tokodaii* XPB–Bax1 complex bound to a forked DNA showed that XPB encircles the duplex–single-strand junction, with its thumb-like motif inserting between the strands while Bax1 binds the opposite arm [48]. Mutations in the RED and ThM motifs impaired DNA binding, and ATPase assays confirmed their requirement for catalytic activity. The data suggests that ATP binding and hydrolysis drive conformational changes that move XPB along the duplex by roughly two base pairs per catalytic cycle. XPB also exhibited stronger affinity for mismatched bubble DNA than for fully paired duplexes, indicating selective engagement of lesion-like DNA structures [48]. Comparison with the human TFIIH–XPA–DNA complex revealed that archaeal XPB occupies an equivalent position at the DNA junction, while Bax1 aligns with XPA at the fork, consistent with a conserved mechanism of bubble propagation and stabilization during NER [48].

### 2.3. Disease-Associated Mutations of XPB

Mutations in the XPB helicase are associated with XP, CS, and TTD, reflecting variable effects on the TFIIH complex. Variants that preserve TFIIH integrity are typically associated with XP, whereas those that compromise complex stability are observed in CS and TTD. Cells carrying these mutations showed reduced NER activity, impaired recovery of RNA synthesis after UV exposure, and lower XPB protein levels. Functional assays indicated that some missense variants retained partial repair capacity, while truncating and splice-site mutations resulted in unstable or truncated proteins, correlating with increased disease severity [49,50,51]. It has also been suggested that XPB dysfunction can cause TFIIH to destabilize and prevent transcription coupled repair, which may enhance the relationship between different molecular changes and disease [12,52].

Mutations in XPB are exceptionally rare compared with other TFIIH subunits because its activity is essential for general transcription, and complete loss of function is incompatible with viability [52]. In cases where the complex remains partially stable, repair-specific defects predominate, whereas mutations that destabilize TFIIH lead to combined deficiencies in repair and transcription, producing the developmental and neurological features of CS and TTD [52].

In XPB, mutation of the conserved lysine residue K346 within the Walker A motif abolishes its ability to bind and hydrolyze ATP, disrupting both transcription and DNA repair [53]. This mutation prevents XPB from localizing to DNA damage sites and renders it incapable of supporting the ATP-driven steps of NER in vitro [54]. Because XPB is required for promoter opening during transcription initiation, these mutations also lower global transcriptional efficiency, contributing to developmental abnormalities observed in XPB-related disorders [55].

To determine the extent to which XPB is somatically altered across human malignancies, a pan-cancer analysis evaluated genomic alterations of 12 DNA helicases in 10,245 tumors spanning 33 cancer types from The Cancer Genome Atlas (TCGA) [56]. Somatic mutation analysis included nonsynonymous variants classified as frame-shift deletion, frame-shift insertion, in-frame deletion, in-frame insertion, missense mutation, nonsense mutation, nonstop mutation, splice site, and translation start site. Across all tumors analyzed, the total mutation frequency for the 12 helicases was 4.5% (464/10,245 tumors). Within this dataset, XPB exhibited a mutation frequency of approximately 2%, placing it among the helicases with the lowest mutation frequencies. Copy number alterations were examined in 9991 tumor samples using GISTIC2.0, where amplification was defined as SCNA (Somatic Copy Number Alteration) value = 2 and deep deletion as SCNA value = −2. XPB was not identified among helicases with predominant amplification or deep deletion events, and gene loss was reported as seldom observed for most helicases. Among the 33 cancer types analyzed, uterine corpus endometrial carcinoma (UCEC) exhibited the highest overall mutation frequency across the 12 helicases collectively [56].

### 2.4. XPB Beyond Transcription and NER

Beyond its canonical roles in transcription and repair, XPB (and XPD) has also been implicated in genome-protective functions such as the degradation of retroviral cDNA, suggesting an additional layer of defense against viral integration [57]. XPB’s involvement with viral genomes also extends to transcriptional regulation, where certain viruses directly exploit its promoter-opening activity [58]. Human T-cell Lymphotropic Virus type 1 (HTLV-1) Tax forms a direct physical interaction with XPB and recruits it to the HTLV-1 long terminal repeat (LTR) promoter, where XPB’s ATPase activity is required for promoter opening. XPB overexpression increases Tax-dependent LTR activation, while XPB depletion or inhibition of XPB with spironolactone suppresses LTR promoter activity; furthermore, an XPB mutant defective in the ATPase activity required for promoter opening does not rescue the inhibitory effect of spironolactone [58]. Tax therefore exploits XPB as an essential host cofactor to drive HTLV-1 transcription [58].

### 2.5. XPB Emerging Research in Cancer Biology and Treatment

XPB is significantly overexpressed in hepatocellular carcinoma (HCC) showing a strong association with poor clinical prognosis [59]. Functional assays in HepG2 cells demonstrated that increased XPB expression enhances cell proliferation and migration, while transcriptomic analyses revealed enrichment of immune-related and inflammatory pathways. Elevated expression levels were identified as an independent prognostic factor for HCC, supporting patient-specific therapeutic approaches and providing a framework for precision immunotherapy guided by tumor molecular characteristics [59].

**Figure 3:**
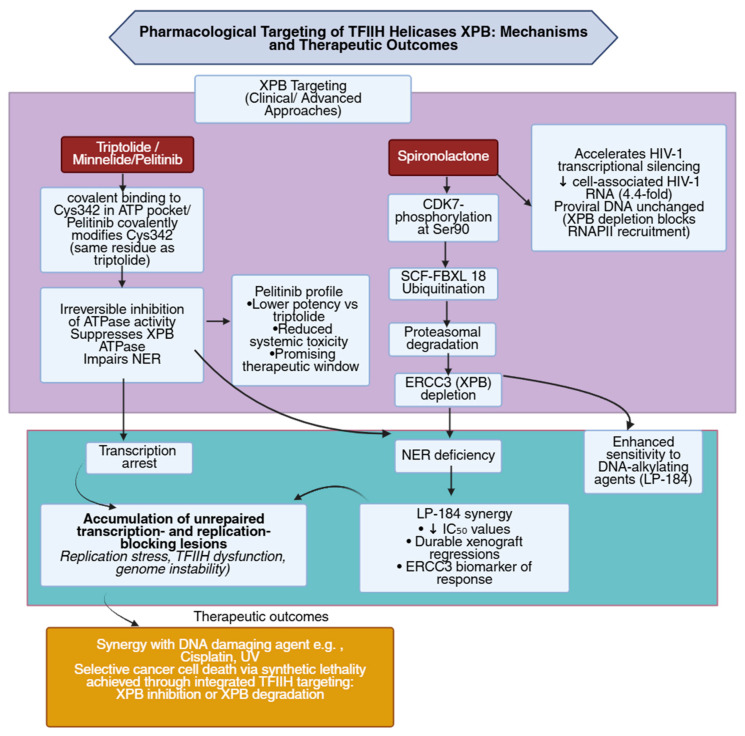
Therapeutic strategies targeting XPB within the TFIIH complex. This schematic diagram summarizes established and experimental approaches to modulating XPB activity in disease processes explained in this review. Clinically explored XPB-directed compounds, including triptolide derivatives and spironolactone, act through covalent inhibition or proteasome-dependent XPB depletion, leading to transcriptional shutdown, impaired NER, and accumulation of DNA damage. These disruptions sensitize cells to DNA-damaging therapies and can promote synthetic lethal killing of cancer cells.

Increasing structural and biochemical evidence has identified XPB as a vulnerable enzymatic target within the TFIIH complex, central to both transcription initiation and NER (Figure 3) [60]. Triptolide forms a covalent bond with a conserved cysteine residue in the XPB ATPase domain, irreversibly blocking ATP hydrolysis and disabling DNA opening at promoter regions [61,62,63]. Inhibition of XPB through this mechanism prevents TFIIH-dependent promoter melting and transcription initiation, resulting in global suppression of RNA synthesis and accumulation of unrepaired DNA lesions that generate synthetic lethality in cancer cells experiencing high transcriptional and replicative stress [60]. Functional studies have shown that XPB operates together with XPD and CDK7 as part of a tri-enzymatic module that coordinates promoter clearance and DNA repair, and selective inhibition of XPB within this complex destabilizes TFIIH assembly and interrupts recruitment of downstream transcription factors, causing transcriptional arrest while largely preserving basal expression of housekeeping genes [64]. Cancer cells, which depend on sustained transcriptional activity, display pronounced sensitivity to XPB inhibition, highlighting its therapeutic potential [60]. XPB is the direct cellular target of triptolide, with mass spectrometry and mutational analyses identifying Cys342 as the covalent modification site responsible for enzyme inactivation and TFIIH destabilization [61].

Pharmacological refinement of triptolide led to the development of minnelide, a water-soluble prodrug with improved stability and delivery, which maintains XPB-targeting activity while demonstrating potent antitumor efficacy across multiple preclinical cancer models [65]. The capacity of minnelide to suppress both transcription and repair underscores the feasibility of XPB inhibition as a strategy for selectively targeting transcriptionally active malignancies [65].

Recently, a new inhibitor of XPB was discovered using a high-content screening (HCS) approach [66]. Pelitinib emerged as an XPB inhibitor that covalently modifies Cys342 of XPB, similar to triptolide. It suppresses XPB’s ATPase activity and impairs NER [66]. Although less potent than triptolide, pelitinib shows lower toxicity and demonstrates promising inhibitory effects on XPB [66].

Spironolactone, a widely used mineralocorticoid receptor antagonist, has been identified as a pharmacological inhibitor of XPB [67]. Experimental studies in keratinocytes and human skin explants showed that spironolactone rapidly depletes XPB protein, suppresses ATR (ataxia telangiectasia and Rad3-related) and ATM (ataxia telangiectasia mutated) kinase activation, blocks removal of UV-induced DNA lesions, and sensitizes cells to UVB cytotoxicity, establishing that XPB depletion pharmacologically mimics a DNA-repair-deficient state associated with elevated mutagenesis and potential carcinogenic risk [68,69]. High-dose spironolactone and canrenone—the primary active metabolite of spironolactone—depleted XPB in keratinocytes and human skin explants and induced replication-stress signaling, apoptosis, and notable epidermal toxicity [70]. Although these effects highlight the need to control local drug levels, spironolactone remains a promising XPB-targeting agent, and improved topical delivery systems are being developed to enable safer and more effective skin-directed use [70,71,72,73].

Biochemical and mechanistic analyses revealed that spironolactone promotes proteasome-dependent degradation of XPB through CDK7-mediated phosphorylation at Ser90 and SCFFBXL18-dependent ubiquitination, impairing NER and transcription initiation while enhancing tumor-cell sensitivity to cisplatin and other DNA-damaging agents. XPB degradation by spironolactone depends on the ubiquitin-selective segregase VCP/p97, which extracts ubiquitinated XPB from holo-TFIIH for proteasomal turnover [74,75]. XPB loss also increased expression of NKG2D (natural killer group 2, member D) ligands, indicating immunomodulatory activity that may strengthen tumor immunosurveillance [67].

Complementary viral studies demonstrated that spironolactone prevents Epstein–Barr virus (EBV) replication by depleting XPB and blocking its recruitment to SM (the EBV early lytic regulatory protein)–dependent lytic promoters, thereby inhibiting transcription of essential viral genes and reinforcing XPB’s broader relevance as a druggable transcription–repair target with implications for cancer and antiviral therapy [76]. Further supporting this XPB-dependent control point, the Hsp90 inhibitor C210 was shown to trigger lytic entry yet prevent completion of the viral program by promoting proteasome-dependent XPB degradation, resulting in selective loss of SM-dependent late-gene expression and a complete block in infectious virion production [77].

Additional work in HIV-1 latency models showed that spironolactone-induced XPB degradation rapidly suppresses HIV-1 transcription by reducing RNA polymerase II recruitment to the viral genome [78]. XPB knockdown produced comparable decreases in HIV-1 RNA and capsid levels, confirming that transcriptional inhibition results from XPB loss [78,79]. Spironolactone enhanced the activity of LP-184, an acylfulvene-derived alkylating agent, by inducing XPB degradation, lowering LP-184 IC_50_ values, and producing more durable xenograft regressions—findings that identify XPB as a biomarker of LP-184 sensitivity and support clinical testing of this combination [80,81]. In vivo studies in HIV-infected humanized mice further demonstrated that spironolactone enhances antiretroviral therapy (ART) by accelerating plasma viremia decay and producing a statistically significant 4.4-fold reduction in cell-associated HIV-1 RNA across tissues, while leaving proviral DNA levels unchanged. This establishes spironolactone as an XPB-targeted transcriptional suppressor capable of strengthening ART-mediated viral silencing [82]. The various roles of XPB presented in this section of the review are summarized in Table 1.

## 3. XPD

XPD is a 5′ to 3′ helicase within the TFIIH complex that drives DNA unwinding for damage verification during NER while helping maintain TFIIH stability during transcription (Figure 1) [86,87]. Its activity is precisely controlled by TFIIH subunits: p44 enhances catalytic efficiency and DNA binding, whereas the CAK kinase module, through its MAT1 component, restrains helicase function during transcription to prevent unnecessary DNA opening [53,88]. These coordinated mechanisms ensure that XPD functions only when required, preserving the balance between repair and transcription.

Mutations disrupting this regulation lead to distinct clinical outcomes. Variants affecting catalytic or DNA-binding residues impair lesion verification and cause XP, while those weakening TFIIH subunit interactions often produce TTD phenotypes [86,88]. Beyond these canonical roles, XPD contributes to broader cellular pathways including mitotic regulation and redox homeostasis, underscoring its importance as a versatile enzyme in genome maintenance and cellular stability [88].

### 3.1. XPD’s Role in Transcription

**Figure 4:**
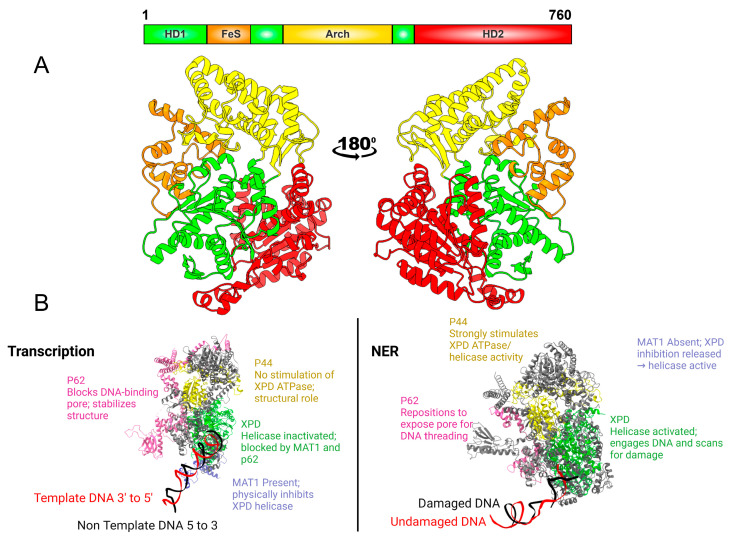
Structure of XPD and changes in its interactions with regulatory partners in TFIIH from transcription to NER. (**A**) Domain organization of human XPD and 3D structure predicted by AlphaFold3. (**B**) Conformational reorganization of TFIIH from transcription to NER. Structural comparison of TFIIH during the transcription in the pre-initiation complex (**left**; PDB: 5OQJ) and NER (**right**; PDB: 7K04) highlights major positional and functional changes in p62 (pink), p44 (yellow), XPD (green), and MAT1 (blue). During transcription, p62 occludes the DNA-binding pore and contributes to structural stabilization of TFIIH, while MAT1 physically restrains XPD, keeping its helicase activity inactive. In this state, p44 plays a predominantly structural role and does not stimulate XPD ATPase activity. Upon transition to NER, MAT1 dissociates, relieving inhibition of XPD, and p44 strongly stimulates XPD ATPase/helicase activity. Concurrently, p62 repositions to expose the DNA-binding pore, enabling damaged DNA engagement. Activated XPD engages the DNA substrate and scans for lesions, facilitating repair bubble formation. DNA strands are color-coded to indicate damaged and undamaged states, with other TFIIH subunits rendered in gray for clarity, emphasizing functional remodeling of TFIIH that licenses XPD helicase activity during DNA repair. For visualization, only XPB, XPD, p44, p62, p52, p8, and DNA were retained; all other proteins were omitted, with MAT1 additionally retained in the PIC structure.

In the TFIIH structure, MAT1 interacts with the ARCH domain on the exterior of XPD through its RING and helical-bundle regions, forming part of the network that includes XPB, p62, and p44 and collectively stabilizes XPD within the core (Figure 4B, left). This binding restricts the flexibility of the ARCH domain needed for substrate loading, thereby keeping XPD in a helicase-inactive configuration compatible with transcription. During NER, dissociation of MAT1 and the CAK complex restores mobility to the ARCH domain and reactivates XPD’s helicase function [11,89].

In addition, Greber et al. demonstrated that XPD engages in a tightly packed interface with p44, forming a regulatory contact that modulates its activity [29]. The XPD–p44 surface area is comparatively small (~940 Å^2^) relative to those of XPD–p62 or XPB–p52, explaining its higher sensitivity to mutations [29]. Disease-associated substitutions, like XPD R722W destabilize this interface by disrupting a salt bridge between XPD and p44 [29]. Such mutations often localize near the periphery of XPD, where they compromise TFIIH assembly and underline the transcriptional defects characteristic of TTD [29].

Cryo-EM structures of the yeast RNA polymerase II pre-initiation complex (PIC) containing TFIIH revealed how the complex is arranged during transcription initiation [25]. The TFIIH core forms a crescent-shaped assembly extending from the translocase Ssl2 to Rad3 (yeast XPD) [25]. Within this structure, Ssl1 and Tfb1, which correspond respectively to the human TFIIH subunits p44 and p62, interact with Rad3 to stabilize its position in the complex [25]. Rad3 is located about 40 Å away from the downstream DNA, consistent with its helicase activity being inactive in transcription [25]. These structural features indicate that XPD adopts a non-catalytic, stabilizing role within TFIIH, where its interactions with Ssl1/p44 and Tfb1/p62 help maintain the overall organization of the TFIIH core in the PIC [25].

Resolved cryo-EM structures of the fully reconstituted dual-incision complex further defined XPD positioning within the pre-incision assembly [40]. In the ATP-bound state, a DNA bubble of approximately 10 base pairs was observed, with XPD engaging 4–9 nucleotides of the unpaired damaged strand. The structures capture XPD bound to the single-stranded segment generated within this bubble while remaining integrated within the assembled repair complex. These data provide structural visualization of XPD associated with the locally unwound DNA substrate within the complete excision machinery, extending prior models by directly resolving the DNA segment accommodated by XPD in the pre-incision state [40].

Genome-wide ChIP-seq analysis in human cells revealed that approximately 40% of XPB and XPD binding sites coincide with predicted G-quadruplex motifs, particularly near transcription-start regions of actively transcribed genes [84]. Biochemical assays showed that XPD unwinds G-quadruplex DNA, whereas XPB binds but does not unwind, indicating complementary roles of the two TFIIH helicases in resolving transcription-associated quadruplexes and preserving genome stability [84]. Consistent with these observations, a genome-wide analysis identified strong enrichment of XPB and XPD binding at promoter-proximal G-quadruplex structures, particularly interstrand G4 motifs, supporting a functional relationship between TFIIH helicases and quadruplex DNA [85].

### 3.2. XPD’s Role in NER

XPD plays a key role in NER for damage verification [1]. Structural modeling of transcription-coupled repair (TCR) shows that TFIIH is initially recruited in an open conformational state in which the two translocase subunits, XPB and XPD, are kept distal, and XPD is expressly sequestered away from substrate DNA [90]. During this early stage, XPD remains helicase-silent until subsequent TFIIH reorganization enables its repositioning and engagement with DNA. XPB-driven DNA unwinding, recruitment of XPA, and movement of XPD transform this inactive arrangement into a repair-competent assembly [90]. Upon NER activation, XPA remodels TFIIH by releasing the kinase module and unblocking a segment that occludes XPD’s DNA-binding pore, enabling the helicase to load onto DNA and initiate strand unwinding [12].

Building on these findings, high-resolution cryo-EM structures of the human XPC–TFIIH–XPA–DNA complex further revealed that XPA bridges XPB and XPD by substituting for the C-terminal helix of XPC, rotating the lesion-containing DNA by roughly one helical turn, and aligning the damaged strand for 3′ → 5′ threading into XPD’s helicase channel during lesion verification [13]. Recruitment of XPA and XPG triggers a major rearrangement of TFIIH that removes the kinase module and opens XPD’s DNA-binding pore. This transition repositions XPB and XPD onto double- and single-stranded DNA, aligning them for their translocase and helicase functions. Through these changes, XPA and XPG jointly activate XPD’s helicase activity and promote its engagement with the repair bubble [12]. This rearrangement shifts XPD and its partner p44 by roughly 80 Å, repositioning the FeS and Arch domains to secure the 3′ single-stranded DNA and begin lesion detection. The large movement depends on flexible linkages between p44 and p34 and on coordinated structural adjustments within p52, which accommodate the repositioning of XPD during repair [12].

Once TFIIH adopts this repair-active configuration, XPA serves as an essential activator that stabilizes the open complex and promotes XPD loading onto damaged DNA. By relieving kinase-mediated repression and supporting the proper alignment of XPD’s DNA-binding elements, XPA enables efficient helicase engagement and lesion scanning [91]. Together with XPG, XPA markedly enhances XPD’s catalytic performance—helicase assays show approximately fourfold stimulation by XPA and twentyfold by XPG—directly linking these structural transitions to functional activation during NER [12].

Combined cryo-EM, cross-linking mass spectrometry, and AlphaFold2-based modeling to build the pre-incision complex, found that bulky DNA lesions stall within XPD’s DNA-binding groove at a constriction point near His135, and that in this stalled position the XPG hydrophobic wedge and β-hairpin motifs could act as a helicase pin to facilitate strand separation during XPD unwinding, with XPD also shown to stimulate XPG incision after completion of lesion scanning [92]. A similar lesion scanning to incision switch has been demonstrated in combined molecular dynamics coupled with single-molecule experiments, in which stalling of XPD at a bulky lesion disrupts its ATP-driven scanning and directly licenses XPG incision [93].

In addition to these canonical repair factors, other cellular proteins can also modulate XPD activity under genotoxic conditions. Ribosomal protein S3 (rpS3) interacts directly with the C-terminal helicase domain of XPD within TFIIH, enhancing helicase activity and promoting complex turnover on UV-damaged DNA [94]. Overexpression of rpS3 in XP-D (R683W) cells, which carry a helicase-defective XPD variant associated with XP, partially restored UV resistance and repair efficiency, whereas rpS3 depletion reduced NER capacity [94]. Although XPD primarily functions in nuclear NER, it has also been detected in mitochondria, where canonical NER is absent. This localization suggests that XPD may participate in noncanonical mitochondrial repair pathways, although their mechanisms remain unclear [95].

XPD’s method of damage recognition can change according to the type of DNA damage [96,97]. Molecular dynamics simulations have been used to compare how human XPD engages the two major UV photolesions, cyclobutane pyrimidine dimer (CPD) and pyrimidine 6−4 pyrimidone (6–4PP) [98]. CPDs remain blocked outside the DNA entry pore and are sensed primarily by external lesion-sensor residues, whereas 6–4PPs can enter the pore and trigger a large Arch-domain displacement that allows the lesion to become tightly accommodated [98]. These findings indicate that XPD verifies CPD and 6–4PP through distinct structural responses, with pore entry and Arch-domain movement contributing specifically to 6–4PP recognition [98].

A major advance in understanding how the XPD helicase recognizes bulky DNA lesions was achieved using cryo-electron microscopy [99]. The study resolved the *Chaetomium thermophilum* XPD–p44–p62 complex bound to a synthetic Y-fork DNA substrate containing an engineered interstrand cross-link at 3.1–3.4 Å resolution, representing a noncanonical NER substrate [99]. The structures revealed that duplex DNA unwinding proceeds until the lesion encounters the Arch domain of XPD, where the cross-linked DNA is physically stalled within the helicase channel. This structural stalling of duplex DNA at the Arch domain defines the point at which XPD halts translocation, illustrating that damage verification arises from the physical architecture of the DNA–protein complex rather than direct chemical recognition of the lesion [99].

Biochemical and mutagenesis analyses showed that all XPD variants displayed DNA-binding affinities and ATPase activities comparable to wild type, indicating that neither property was substantially affected [99]. However, helicase activity varied markedly among the mutants: W373A exhibited enhanced unwinding (172% of wild type), W372E showed a slight reduction (88%), and both R372A and R372E displayed a strong decrease (16% and 19%, respectively), demonstrating that R372 is highly relevant for duplex separation [99]. The cryo-EM maps showed no density for p62 or the C-terminal region of p44, indicating that these components were not resolved in the reconstruction [99]. These findings suggest a dynamic regulatory response within TFIIH upon damage verification. Collectively, these findings establish XPD as a lesion verification factor within the NER machinery, operating as a kinetic and structural checkpoint that safeguards incision fidelity by ensuring that only bona fide helix-distorting lesions are processed by downstream nucleases such as XPG [99].

Furthermore, using recombinant *Chaetomium thermophilum* homologs, the authors demonstrated that XPD’s helicase and ATPase activities are significantly enhanced when bound to the full-length p44/p62 heterodimer, compared to when complexed with the traditional truncated N-terminal p44 fragment (N-p44) [100]. While N-p44 alone provides the basal stimulation of XPD’s ATPase function, inclusion of p62 further amplifies this activity by increasing XPD’s affinity for double-stranded DNA by roughly threefold, and by an extraordinary ~60-fold in the presence of damaged DNA. Kinetic measurements showed that this enhancement arises primarily from a decrease in the apparent K_m_ (indicating tighter substrate binding) rather than a change in the catalytic turnover k_cat_. Thus, p62 acts as an allosteric enhancer, stabilizing XPD in a conformation optimized for interaction with damaged DNA substrates [100]. In contrast, XPD–N-p44 shows similar affinities for damaged and undamaged DNA, underscoring that the damage-specific recognition originates from the presence of p62 rather than p44 alone [100].

Cryo-EM modeling provided a structural explanation for these biochemical results. In the apo-TFIIH structure, p62 occupies the DNA-binding channel of XPD, likely preventing DNA entry in the absence of substrate [100]. However, in DNA-bound TFIIH, p62 relocates toward the Arch domain, where it interacts with both XPD and the single-stranded DNA passing through its helicase cleft. This repositioning likely alters the FeS-cluster–containing pocket that guides DNA translocation and may facilitate lesion verification during NER. Collectively, these findings position XPD as a dynamically regulated helicase, whose DNA damage affinity and catalytic activity are fine-tuned by its association with p44 and p62. Rather than serving as static structural elements, p44/p62 function as regulatory cofactors that sensitize XPD to DNA damage, thereby coupling its helicase function directly to lesion recognition during the initial steps of NER [100].

Single-molecule magnetic-tweezers analysis of isolated human XPD revealed intrinsically slow and low-processivity helicase activity, with unwinding rates of approximately 0.3 bp s^−1^ and average event lengths of ~14 bp. The slow rate reflects an inherently sluggish ssDNA translocation rather than impaired strand separation, and the enzyme spends much of its time paused or inactive. Compared with archaeal and TFIIH-bound XPD, the isolated human enzyme is 40–60 times slower, emphasizing that cofactors such as p44 and p62 are essential for achieving full catalytic efficiency [101].

Biochemical studies have elucidated how XPD and its regulatory partner p44 interact with DNA containing structurally distinct bulky lesions [102]. Through fluorescence anisotropy, ATPase, and photo–cross-linking assays it was shown that XPD–p44 binding affinity increases with lesion size and repairability, correlating with the known NER excision efficiencies. Binding strength rose in the order undamaged < Fap-dC (unrepairable) < nAnt < nFlu (highly repairable), with nFlu DNA, showing a ~166-fold greater affinity than undamaged DNA. While ATPase activity of XPD–p44 changed little between damaged and undamaged substrates, photo-cross-linking demonstrated that both XPD and p44 form covalent adducts with damaged single-stranded regions, enhanced by ATP and by p44 itself. These results indicate that lesion recognition and verification depend primarily on the structural nature of the damage and the affinity of XPD–p44 rather than on ATP hydrolysis efficiency. Importantly, p44 was shown to directly engage damaged DNA, suggesting it functions not merely as a helicase activator but as a co-sensor during damage verification. Collectively, the study supports a model in which efficient NER relies on structural discrimination of lesions by the XPD–p44 subcomplex, establishing p44 as an integral participant in verifying bulky DNA adducts and highlighting that the damage-dependent modulation of XPD–p44 interaction underlies the variable repair efficiencies observed across different lesion types [102].

### 3.3. Diseases Associated with XPD Mutants

Computational analyses categorized disease mutations in XPD by phenotype: XP mutations localized to the DNA-binding interface and the Walker motifs, CS mutations mapped to computationally identified dynamic communities, and TTD mutations clustered at protein–protein interfaces with other TFIIH subunits such as p44 [103]. Additional structural mapping showed that tumor-associated variants distribute across the RecA1–RecA2 interface, DNA-binding regions, and protein-interaction surfaces within XPD [103].

Recent findings have identified an immunological consequence of XPD loss, extending its role beyond NER to transcriptional regulation [104]. In three trichothiodystrophy (TTD1) patients carrying compound heterozygous ERCC2 variants—including two novel truncations—XPD protein was markedly reduced and cells exhibited classical NER failure. Upon UV irradiation, patient lymphoblastoid cells showed elevated γ-H2AX accumulation, confirming persistent DNA lesions due to defective TFIIH-mediated unwinding. Transcriptomic analysis revealed down-regulation of EGR1–3, early transcription factors required for B-cell receptor (BCR)–driven activation, and reduced expression of immunoglobulin heavy-chain genes (IGHM, IGHD, IGHG1-4). Flow cytometry confirmed decreased naïve and transitional B-cell subsets and impaired induction of activation markers CD69 and CD86 after BCR stimulation, while CD40-mediated activation remained intact. These findings implicate XPD specifically in transcription-coupled gene activation, not receptor signaling itself. In discussion, the authors propose that defective XPD helicase activity destabilizes TFIIH, preventing efficient promoter opening and RNA-polymerase II transcription of early immune-response genes, thereby linking genomic maintenance with adaptive-immune competence. Although complementation experiments are still needed to prove causality, in silico prediction algorithms (PHRED, REVEL) classified the new XPD variants as pathogenic, supporting their role in the observed transcriptional and immunological defects [104].

A clinical study described a child with TTD resulting from compound-heterozygous splice-site variants in ERCC2 (c.2190 + 1delG and c.1479 + 2dupT) [105]. The patient exhibited the characteristic clinical features of TTD together with progressive diffuse white-matter hypomyelination observed on serial brain MRI over 4.5 years. In their discussion, the authors noted that only four previous TTD cases with XPD variants had shown myelination abnormalities, none demonstrating progression beyond early childhood. Analysis of publicly available single-cell RNA-sequencing databases indicated that XPD is broadly expressed in neurons, fibroblasts, and mesenchymal lineages throughout the human brain, supporting the gene’s importance in central-nervous-system development. Together, these data directly link XPD deficiency to a progressive hypomyelinating leukodystrophy phenotype, thereby expanding the recognized neurological spectrum of XPD-associated trichothiodystrophy [105].

Extending beyond the progressive leukodystrophy phenotype, new findings now demonstrate that pathogenic XPD variants impair TFIIH-dependent processing of transcription-associated R-loops, providing further mechanistic context for TTD pathology [106]. Pathogenic XPD variants associated with TTD destabilize the TFIIH complex by weakening the connection between its core subunits and the CAK subcomplex, leading to reduced chromatin-bound TFIIH. This imbalance disrupts a larger chromatin-associated assembly containing TFIIH, RNA polymerase II, and the R-loop–processing factors ATP-dependent RNA helicase Dead-Box-helicase 1 (DDX1), splicing factor proline- and glutamine-rich (SFPQ), and non-POU domain–containing octamer-binding protein (NONO), resulting in accumulation of RNA:DNA hybrids. Primary trichothiodystrophy fibroblasts show increased association of these factors with the limited remaining TFIIH and pronounced R-loop buildup, demonstrating that XPD-dependent TFIIH integrity is essential for proper R-loop processing [106].

XPD-mutated cells also show pronounced hypersensitivity to oxidative stress and impaired repair of oxidized DNA lesions, indicating that XPD dysfunction compromises additional genome-maintenance pathways beyond UV-induced NER [107]. This oxidative-stress involvement is further reflected in bupivacaine-treated neuronal cells—bupivacaine being a widely used local anesthetic—where oxidative DNA damage is accompanied by increased expression of XPD and PARP1 [108]. The two proteins show detectable interaction after treatment, and suppression of XPD reduces PARP1 levels, whereas suppression of PARP1 does not affect XPD. Inhibiting either protein increases oxidative DNA damage, and the authors note that defining the interaction between XPD and PARP1 may clarify mechanisms of DNA repair and may represent a potential target for preventing oxidative DNA damage, including bupivacaine-induced injury [108].

A rare clinical case of adenoid cystic carcinoma of the head and neck harboring a germline XPD mutation together with a somatic BRCA2 mutation, in the absence of MYB or MYBL1 gene fusions [109]. Whole-exome sequencing revealed a frameshift mutation in ERCC2, c.578del; p.F193Sfs*55, located within exon 7 of the helicase ATP-binding domain, producing an alteration at residue 193 that may affect protein function. This variant was identified as germline, with an allelic frequency of 48.3%, and was not present in public databases such as gnomAD. This case represents the first documentation of a germline XPD variant in adenoid cystic carcinoma, expanding the molecular spectrum of the disease. The authors conclude that identifying such variants contributes to defining the DNA-damage-repair gene landscape of MYB/MYBL1-fusion–negative tumors [109].

Whole-exome sequencing of pretreatment tumors from 50 patients with muscle-invasive urothelial carcinoma treated with neoadjuvant cisplatin-based chemotherapy identified nine non-synonymous somatic mutations in XPD [110]. XPD was the only gene significantly enriched in cisplatin responders compared with non-responders, and all XPD non-synonymous mutations occurred exclusively in cisplatin-sensitive tumors. The mutation frequency within the responder cohort was 36%, significantly higher than in unselected bladder cancer cohorts, whereas across 19 additional tumor types XPD mutations were observed at low frequencies (<4%). Functional testing demonstrated that representative XPD mutants failed to rescue cisplatin sensitivity in an XPD-deficient cell line, supporting the conclusion that somatic XPD mutation status may inform the use of cisplatin-containing regimens in muscle-invasive urothelial carcinoma [110].

### 3.4. XPD as a Perspective Anti-Cancer Target and Potential Biomarker

**Figure 5:**
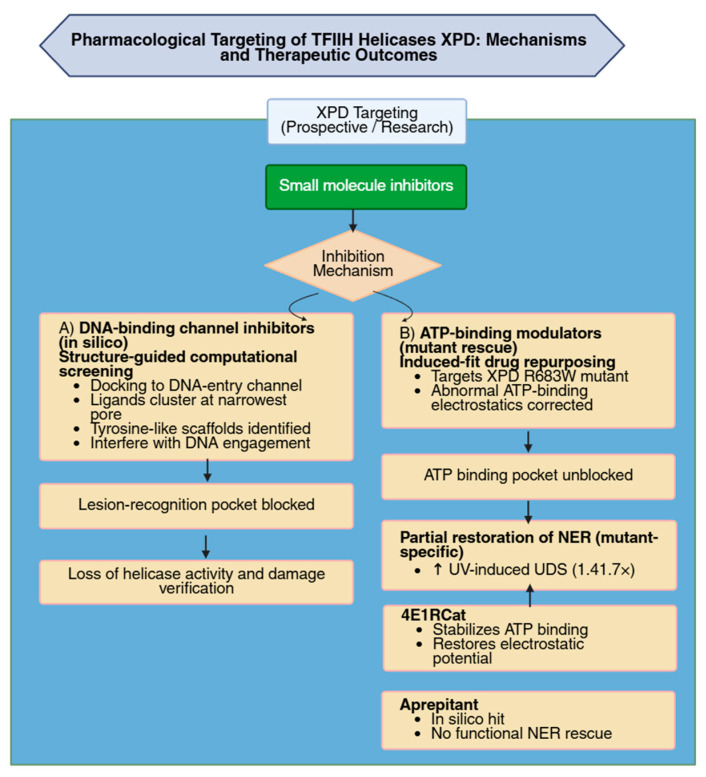
Therapeutic strategies targeting XPD within the TFIIH complex. XPD-directed strategies remain at the research stage and focus on small-molecule inhibition of catalytic or DNA-interacting pockets to block helicase activity and lesion verification. Together, these approaches highlight the therapeutic potential of selectively targeting TFIIH helicases to exploit vulnerabilities in tumor DNA repair and transcription programs.

The XPD helicase within TFIIH functions exclusively in NER, where its enzymatic activity is required for DNA unwinding and lesion verification during the repair process [86]. Because XPD’s catalytic function is confined to DNA repair rather than transcription, it represents an attractive cancer drug target, as inhibition can selectively impair repair of genotoxic damage while leaving general transcription largely unaffected (Figure 5) [64]. Currently, no lead compounds are available that directly target XPD, but recent progress in structural and functional analysis has increased understanding of how XPD operates within TFIIH and may support future development of inhibitors. XPD activity is regulated positively by the TFIIH subunits p44 and p62, and negatively by MAT1, which binds to the Arch domain and blocks the region essential for helicase function [64]. This domain is mechanistically required for helicase activity, and single-point mutations in the Arch domain result in complete loss of function. Based on existing structural data, the ATP-binding site of XPD represents a potential druggable pocket. In the bacterial XPD homolog DinG, a lesion-recognition pocket has been identified [64,111], and the authors note that such a site could be targeted by DNA-damage analogues to interfere with XPD’s repair activity. Somatic tumor mutations which inactivate XPD helicase function are associated with a positive response to cisplatin therapy in urothelial cancers, supporting the concept that XPD impairment enhances chemotherapy sensitivity [64]. Furthermore, mutations in NER genes, including XPD, are linked to improved responses to immune-checkpoint inhibitor therapy [64].

Computational screening framework designed to identify potential small-molecule inhibitors of screening was recently employed against XPD [112]. As part of this analysis, the authors examined XPD by directing docking toward its DNA-binding region. The results showed that compounds consistently clustered within the narrowest part of the DNA-entry channel, revealing several chemotypes capable of occupying this site. One tyrosine-like scaffold was highlighted because the authors note that it may mimic features associated with phosphotyrosyl DNA-adduct interactions. Overall, the screening defines ligand-accessible positions along this DNA-binding path and identifies chemical scaffolds that could interfere with DNA engagement and thus suppress TFIIH and NER [112].

An induced-fit, in silico drug-repurposing approach was used to identify compounds capable of improving ATP binding in the XPD R683W mutant, a pathogenic ERCC2 allele associated with reduced NER [113]. Structural modeling and molecular dynamics analyses showed that the ATP-binding region of XPD R683W exhibits an abnormal positive electrostatic charge relative to wild-type XPD. Screening of 2006 bioactive compounds followed by induced-fit refinement identified 4E1RCat as the strongest candidate based on docking stability and increased frequency of correct ATP-binding poses. Binding of 4E1RCat shifted the electrostatic potential of the mutant ATP-binding region toward a wild-type–like negative state. In primary fibroblasts carrying the R683W mutation, 4E1RCat increased UV-induced unscheduled DNA synthesis by approximately 1.4–1.7-fold relative to solvent controls, indicating partial recovery of NER. Aprepitant, another compound selected during screening, did not improve repair capacity despite its in silico performance [113]. Figure 5 summarizes the current research and therapeutic strategies that target XPD.

A clinical cohort study investigated whether variation in the XPD Lys751Gln single nucleotide polymorphism (rs13181) influences oxaliplatin-related toxicities in colorectal cancer patients receiving CAPOX (capecitabine plus oxaliplatin) chemotherapy [114]. The Lys751Gln substitution has been associated with reduced DNA repair efficiency, which may affect sensitivity to platinum-based chemotherapy. In a prospective cohort of 72 patients receiving the CAPOX regimen, the authors performed PCR-based genotyping followed by Sanger sequencing and monitored both hematological toxicities (anemia, neutropenia, thrombocytopenia) and non-hematological toxicities (peripheral neuropathy, nausea, vomiting, diarrhea) over four chemotherapy cycles. Statistical analysis showed no significant relationship between the XPD Lys751Gln genotype and either hematologic or non-hematologic adverse effects. The study concluded that, within this Iraqi cohort, the XPD Lys751Gln polymorphism does not predict oxaliplatin-induced toxicity, indicating that this genetic variant is unlikely to serve as a reliable pharmacogenetic biomarker for CAPOX-related side effects [114]. The lack of association between Lys751Gln and oxaliplatin-related toxicities may be attributed to the relatively small sample size, single-center recruitment, and limited follow-up of only four cycles, which may reduce the ability to detect genotype–toxicity relationships. Differences in oxaliplatin regimens, dose intensity, treatment duration, and ethnic background across studies may also contribute to inconsistent findings. Collectively, these factors may explain why Lys751Gln did not demonstrate predictive value for toxicity in this cohort [114].

The MIAT/miR-29a-3p/COL4A1 axis is a regulatory pathway in which the long non-coding RNA MIAT suppresses the tumor-suppressive microRNA miR-29a-3p, leading to increased expression of COL4A1, a collagen subunit associated with enhanced migration, invasion, epithelial–mesenchymal transition (EMT), and metastatic progression in hepatocellular carcinoma (HCC) [115,116,117]. This study showed that XPD activates the tumor suppressor protein P53, leading to repression of MIAT and restoration of miR-29a-3p–mediated control of COL4A1, thereby reducing migration, invasion, EMT, and metastatic behavior in HCC models [115]. These findings indicate that XPD influences this pro-metastatic regulatory axis and may represent a potential therapeutic target in HCC [115]. At the molecular level, a separate study has shown that XPD is also regulated through RNA methylation mechanisms in HCC. Nucleolar Protein 2 (NOP2) functions as an m^5^C (5-methylcytosine) RNA methyltransferase, and m^5^C —mediates m^5^C methylation of XPD mRNA, increasing its stability and suppressing malignant progression of HCC. Through analyses of patient datasets and experiments in the human HCC cell line SMMC-7721, the authors demonstrated that both NOP2 and XPD are downregulated in HCC tissues, and that forced NOP2 overexpression increases m^5^C methylation of XPD, elevates XPD expression, and reduces cell proliferation, migration, and invasion [117]. These findings support XPD as a potential therapeutic target in HCC.

Outside of HCC, XPD levels have been evaluated in other patient populations. In end-stage renal disease (ESRD) patients undergoing hemodialysis, XPD expression in blood lymphocytes was significantly reduced compared to healthy controls, whereas miR-145 and miR-770 levels were elevated [118]. Both microRNAs are established negative regulators of XPD, and their increased expression corresponded with lower XPD levels. Hemodialysis altered these expression patterns, particularly increasing XPD and miR-770 in patients without cancer. These findings indicate that reduced XPD expression and increased regulatory microRNAs are features of ESRD, and such expression changes may serve as biomarkers—measurable biological indicators that reflect disease-related alterations in cellular processes [118].

Research has also assessed XPD behavior in esophageal squamous cell carcinoma (ESCC) models. ESCC remains difficult to treat with surgery, chemotherapy, or radiotherapy, underscoring the need to better define its molecular determinants [119]. In this study, XPD expression was reduced in ESCC tissues, and restoring XPD in the EC9706 and EC109 ESCC cell lines suppressed proliferation, migration, and invasion, increased apoptosis, and enhanced sensitivity to cisplatin and fluorouracil. XPD overexpression decreased phosphoinositide 3-kinase (PI3K), phosphorylated AKT (p-AKT), cellular Myc proto-oncogene (c-Myc), Cyclin D1, B-cell lymphoma 2 (Bcl-2), vascular endothelial growth factor (VEGF), and matrix metalloproteinase-9 (MMP-9), changes the authors describe as inhibition of the PI3K/AKT pathway associated with reduced tumor cell growth and survival. XPD also increased p21, which the paper identifies as a regulator of cell-cycle progression and apoptosis whose elevation is linked to decreased proliferation and increased apoptosis. Overall, the findings support XPD as an inhibitor of ESCC growth and invasion and as a potential target for improving chemotherapeutic response [119].

A separate line of investigation has characterized the impact of XPD expression in malignant melanoma cells [120]. The full-length XPD gene was cloned into a eukaryotic expression plasmid and successfully expressed in A375 malignant melanoma cells, as verified by fluorescence from the XPD–EGFP fusion and by Western blot analysis. Immunofluorescence demonstrated that XPD localized to the endoplasmic reticulum. Functional assessment using a 3-(4,5-dimethylthiazol-2-yl)-2,5-diphenyltetrazolium bromide (MTT) assay—an assay that measures metabolic activity as an indicator of proliferative capacity—showed that cells transfected with XPD exhibited significantly reduced metabolic activity compared to control cells. These findings indicate that exogenous XPD expression suppresses proliferation in malignant melanoma A375 cells and provide a basis for further investigation of XPD function in melanoma [120].

### 3.5. XPD’s Non Canonical Roles

Beyond Transcription and NER, XPD has also been identified in roles outside of its canonical roles. A conserved function of ERCC2 in regulating the expression of stress-responsive caspases has been investigated [121]. XPD acts as a transcriptional activator of caspase genes in both *Caenorhabditis elegans* and human cells, independent of its canonical role in apoptosis. In *C. elegans*, the XPD homolog XPD-1 was shown to be required for the stress-induced transcription of the caspase gene ced-3 through an upstream transcription-factor-rich DNA element termed the uTF region. Deletion of this region or loss of XPD-1 impaired ced-3 induction after ultraviolet exposure and reduced organismal survival. Conversely, disruption of XPD-1 or the uTF region enhanced resistance to endoplasmic reticulum and osmotic stress, indicating a differential role of XPD-regulated caspase expression in genotoxic versus non-genotoxic stress [121].

Beyond caspase regulation in *C. elegans*, additional model-organism studies have shown that XPD also contributes to tissue growth and survival through interactions with other cellular factors [122]. Genetic and biochemical analyses in Drosophila showed that XPD functions together with the cytosolic iron–sulfur assembly factor Ciao1 to control organ growth, cell survival, and cell-cycle progression. Loss of XPD produced reduced eye and wing growth, increased apoptotic death, and decreased levels of Cyclin E and Drosophila inhibitor of apoptosis 1 (Diap1), matching the defects seen in Ciao1-deficient tissues. Physical interaction between XPD and Ciao1 was confirmed by GST pull-down and co-immunoprecipitation assays, and each gene’s reduction produced similar phenotypes that were not enhanced by combined knockdown, indicating functional action in the same pathway. Overexpression of XPD rescued the growth defects caused by Ciao1 knockdown, and Ciao1 overexpression likewise rescued XPD depletion. Cyclin E overexpression restored growth in both XPD and Ciao1-deficient backgrounds, and Diap1 overexpression partially rescued Ciao1 loss but not XPD loss. These findings indicate that XPD maintains Cyclin E and Diap1 levels to support proliferation and survival during Drosophila organ development [122]. The different roles of XPD that have been discussed are summarized in Table 2.

## 4. Conclusions

XPB and XPD are key helicases that power TFIIH through processes essential for DNA repair, transcription, and genome maintenance. Their coordinated actions link DNA unwinding with damage recognition and transcriptional control, while defects in either enzyme lead to disorders such as XP, TTD, CS and related syndromes. These helicases also play broader roles in stress response and cellular regulation, underscoring their importance beyond canonical repair and transcription. Future research will orient to better understand how XPB and XPD function across different pathways and how their activities can be modulated in disease and healthcare.

## Figures and Tables

**Table 1:** XPB: Pathways, Biological Roles, Diseases, and Outcomes.

Biological Process/Pathway/Disease	XPB Role in Process	Outcome/Biological Consequence	Refs.
Transcription initiation	ATP-dependent dsDNA translocase; reels downstream DNA; generates torsional strain for promoter opening	Promoter melting + transcription start-site opening	[23,24,25,26,27,28,83]
NER	5′ → 3′ translocase; initiates DNA opening at lesion; works with p52/p8 and XPA to unwind downstream DNA	Formation of repair bubble; prepares DNA for XPD lesion verification	[19,34,35,36,37,38,39]
Regulation by p52/p8	p52/p8 stimulate and limit XPB ATPase; maintain XPB stability	Controlled DNA unwinding; defective interaction causes repair failure	[19,29,30,35,36,37]
Stimulation by XPA	XPA clamps DNA to increase XPB unwinding processivity	Enhanced bubble propagation during NER	[37,38]
Viral genome defense (retroviral cDNA degradation)	Participates in degradation of retroviral cDNA	Protects genome from viral integration	[57]
HTLV-1 infection	Directly binds Tax; Tax recruits XPB to LTR promoter	XPB ATPase enables viral transcription activation	[58]
HIV-1	XPB depletion reduces RNAPII recruitment; spironolactone blocks HIV reactivation	Suppresses HIV transcription/reactivation	[78,79,82]
EBV lytic cycle	Required for SM-induced promoter activation	Loss of XPB prevents EBV lytic gene expression	[76,77]
G-quadruplex DNA	Binds G4 structures (but does not unwind)	Supports transcription at G4-rich promoters	[84,85]
Cancer: HCC (XPB overexpression)	XPB upregulation increases proliferation/migration	Poor prognosis; oncogenic role	[59]
Cancer therapeutics: triptolide	Covalent inhibitor at Cys342 → blocks XPB ATPase	Global transcriptional shutdown; NER inhibition	[60,61,62,63]
Cancer therapeutics: minnelide	Water-soluble triptolide prodrug targeting XPB	Anti-cancer efficacy across models	[65]
Cancer sensitization by spironolactone	Induces proteasomal degradation of XPB	Sensitizes tumors to cisplatin; reduces NER capacity	[67,68,69,70,72,73,74,75]
TFIIH mutations → XP/CS/TTD	Missense or truncating variants reduce XPB stability or ATPase activity	Combined defects in NER and transcription; disease severity varies	[49,50,51,52,53,54]
Archaeal XPB–Bax1 complex	XPB interacts with Bax1 nuclease; dual DNA unwinding and cleavage	Evolutionary mechanism of repair; two kinetic modes (“molecular wrench”)	[41,42,43,44,45,46,47,48]

**Table 2:** XPD: Pathways, Biological Roles, Diseases, and Outcomes.

Biological Process/Pathway/Disease	XPD Role in Process	Outcome/Biological Consequence	Refs.
Nucleotide Excision Repair	5′ → 3′ helicase; lesion scanning & verification; works with p44/p62, XPA, XPG	Damage verification checkpoint; activation of incision	[12,13,86,92,99,100,102,123]
Transcription (non-catalytic)	Structural stabilizer; helicase inactive due to CAK/MAT1	Maintains TFIIH architecture in PIC; not involved in DNA unwinding	[11,25]
Regulation by CAK/MAT1	MAT1 blocks Arch domain → suppresses helicase	Prevents unwinding during transcription	[11,12,89,123]
Activation by p44/p62	p44 increases ATPase; p62 enhances dsDNA and damaged DNA binding	~60× increased damage affinity; full helicase activation	[100,101,102]
Mitochondrial localization	Present in mitochondria (noncanonical repair role)	Possible involvement in mtDNA maintenance	[95]
G-quadruplex DNA	Unwinds G4 structures	Prevents transcriptional stalling at G4-rich promoters	[84,85]
Oxidative stress response	Interacts with PARP1; required for limiting ROS damage	Loss increases oxidative lesions	[107,108]
Caspase regulation	Activates caspase transcription via uTF region (*C. elegans* + human homologs)	Controls stress-induced apoptosis pathways	[121]
Immune dysfunction (B-cell defects)	XPD loss reduces EGR1–3 + Ig heavy-chain transcription	Impaired B-cell activation responses	[104]
TTD, XP, XP/CS	Mutation-dependent effects on TFIIH stability or helicase activity	NER defects, transcription defects, neurological issues	[29,86,88,94,103,104,105,106]
Leukodystrophy (progressive hypomyelination)	ERCC2 splice mutations reduce XPD protein	Progressive CNS myelination defects	[105]
Cancer: NER deficiency → cisplatin sensitivity	Tumor mutations in XPD reduce helicase activity	Enhanced response to platinum therapy	[64]
Cancer: HCC (MIAT/miR-29a-3p axis)	Activates P53 → suppresses MIAT → increases miR-29a-3p	Blocks EMT, invasion, metastasis	[115,116,117]
Adenoid cystic carcinoma	Germline ERCC2 frameshift	Expands tumor mutation spectrum	[109]
DNA damage checkpoint (cross-linked DNA)	Arch domain stalls on bulky lesions	Helix-distortion-based verification	[99]
R-loop processing	TFIIH-linked helicase activity helps resolve or prevent transcription-associated R-loop structures.	Limits transcription-associated DNA damage and supports genome stability	[106]

## Data Availability

No new data were created or analyzed in this study.

## Abbreviations

ATP, adenosine triphosphate; ATR, ataxia telangiectasia and Rad3-related kinase; ATM, ataxia telangiectasia mutated kinase; BER, base excision repair; CAK, CDK-activating kinase; CDK7, cyclin-dependent kinase 7; ChIP-seq, chromatin immunoprecipitation sequencing; CS, Cockayne syndrome; CSA, Cockayne syndrome group A protein; CSB, Cockayne syndrome group B protein; DDR, DNA damage response; DNA, deoxyribonucleic acid; dsDNA, double-stranded DNA; EBV, Epstein–Barr virus; EMT, epithelial–mesenchymal transition; ERCC1, excision repair cross-complementation group 1; ERCC2 (XPD), excision repair cross-complementation group 2; ERCC3 (XPB), excision repair cross-complementation group 3; ERCC4 (XPF), excision repair cross-complementation group 4; ERCC5 (XPG), excision repair cross-complementation group 5; FeS, iron–sulfur (cluster/domain); GG-NER, global genome nucleotide excision repair; G4, G-quadruplex; HCC, hepatocellular carcinoma; HIV, human immunodeficiency virus; Hsp90, heat shock protein 90; kDa, kilodalton; LTR, long terminal repeat; MAT1, ménage à trois 1 (CAK subunit); MIAT, myocardial infarction–associated transcript; NER, nucleotide excision repair; NKG2D, natural killer group 2, member D; NOP2, Nucleolar Protein 2; nt, nucleotide; PARP1, poly(ADP-ribose) polymerase 1; PIC, pre-initiation complex; Pol, DNA polymerase; RNA, ribonucleic acid; RNAPII, RNA polymerase II; ROS, reactive oxygen species; rpS3, ribosomal protein S3; SCNA, Somatic Copy Number Alteration; SF2, superfamily 2 helicases; ssDNA, single-stranded DNA; TCGA, The Cancer Genome Atlas; TC-NER, transcription-coupled nucleotide excision repair; TFIIE, transcription factor II E; TFIIH, transcription factor II H; TTD, trichothiodystrophy; UCEC, uterine corpus endometrial carcinoma; UV, ultraviolet; m^5^C, 5-methylcytosine; UVB, ultraviolet-B radiation; VCP/p97, valosin-containing protein; XP, xeroderma pigmentosum; XPA, xeroderma pigmentosum group A protein; XPB, xeroderma pigmentosum group B helicase; XPC, xeroderma pigmentosum group C protein; XPD, xeroderma pigmentosum group D helicase; XPF, xeroderma pigmentosum group F endonuclease; and XPG, xeroderma pigmentosum group G endonuclease.
